# Neutrophil-Lymphocyte Ratio and Circulating Tumor Cells Counts Predict Prognosis in Gastrointestinal Cancer Patients

**DOI:** 10.3389/fonc.2021.710704

**Published:** 2021-07-07

**Authors:** Chengcheng Qian, Renjie Cai, Wenying Zhang, Jiongyi Wang, Xiaohua Hu, Yanjie Zhang, Bin Jiang, Haihua Yuan, Feng Liu

**Affiliations:** Department of Oncology, Shanghai Ninth People’s Hospital, Shanghai Jiao Tong University School of Medicine, Shanghai, China

**Keywords:** neutrophil–lymphocyte ratio, circulating tumor cells counts, prognosis, gastrointestinal cancer, survival

## Abstract

**Purpose:**

The purpose of this study is to explore the prognostic value of associating pre-treatment neutrophil–lymphocyte ratio (NLR) with circulating tumor cells counts (CTCs) in patients with gastrointestinal cancer.

**Materials and Methods:**

We collected the related data of 72 patients with gastric cancer (GC) and colorectal cancer (CRC) who received different therapies from August 2016 to October 2020, including age, gender, primary tumor location, TNM stage, tumor-differentiation, NLR, CTCs, disease-free survival (DFS) and overall survival (OS). We chose the optimal cut-off value of NLR >3.21 or NLR ≤3.21 and CTC >1 or CTC ≤1 by obtaining receiver operating characteristic (ROC) curve. The Kaplan–Meier survival analysis and Cox regression analysis were used to analyze DFS and OS. To clarify the role of the combination of NLR and CTCs counts in predicting the prognosis, we analyzed the DFS and OS when associated NLR and CTCs counts.

**Results:**

A high NLR (>3.21) was associated with shorter DFS (P <0.0001) and OS (P <0.0001). Patients with high CTCs level (>1) had shorter DFS (P = 0.001) and OS (P = 0.0007) than patients with low CTCs level. Furthermore, patients who had both higher NLR and higher CTCs counts had obvious shorter DFS (P <0.0001) and OS (P <0.0001).

**Conclusions:**

Patients with higher NLR and more CTCs respectively tended to have poor prognosis with shorter DFS and OS, which might be regarded as predictors of gastrointestinal cancer. In particular, associating NLR and CTCs counts might be a reliable predictor in patients with gastrointestinal cancer.

## Introduction

Gastrointestinal cancer is a kind of primary tumor located in the digestive tract, which mainly includes gastric cancer (GC) and colorectal cancer (CRC). GC and CRC are the most common malignancies in the world. The 5-year survival rate was approximately 30% in GC patients with radical surgery (1) and about 68.9% in CRC postoperative patients (2). The prognosis of GC and CRC patients was significantly correlated with tumor location, TNM stage, and the tumor size (3). The patients after surgery are mainly monitored by imaging examinations for recurrence or metastasis, but imaging is difficult to detect micro-metastatic lesions. Therefore, a sensible and easy indicator is urgently required to assist predicting prognosis of GC and CRC, which is significant for improving survival.

Nowadays, increasing studies have been suggesting that chronic inflammation is closely related to tumorigenesis and development. Inflammation is one of the key components of tumor microenvironment (TME), which is mainly comprised of tumor cells, stroma cells and immune cells (4). The prognosis of malignancies appeared to be predicted by a variety of inflammatory indices such as neutrophil–lymphocyte ratio (NLR), platelet–lymphocyte ratio (PLR), monocyte–lymphocyte ratio (MLR), systemic immune-inflammatory index (SII), systemic inflammatory marker (SIM) and so on. There were several studies that indicated that NLR as an immunoinflammatory marker can predict the prognosis of certain tumors, such as breast cancer, non-small-cell lung cancer, head and neck squamous cell carcinoma, gastric cancer, and colorectal cancer (5–10).

Circulating tumor cells (CTCs) are kinds of tumor cells that enter into the bloodstream from a primary or metastatic tumor location that may have metastatic potential. Currently, CTCs have been reported to have prognostic value in breast cancer, non-small-cell lung cancer, head and neck squamous cell carcinoma, gastric cancer and colon cancer (11–17).

The prognostic values of NLR (5–10) and CTCs (11–17) were studied respectively. In our former studies about CTCs, we observed that in advanced colorectal cancer patients, CTCs could be a predictor for prognosis, and more CTCs tended to accompany with shorter survival (17). Similarly, in patients with head and neck squamous cell carcinoma, it was also obvious that a high CTCs level was associated with poor prognosis (18). Additionally, we also come to a conclusion that SIM positively correlated with tumor progression in head and neck squamous cell carcinoma patients (14). With the consideration of CTCs and inflammatory index having both a role in predicting the prognosis of tumors, our study not only demonstrated the NLR and CTCs as factors to predict the prognosis, but we also intended to combine the NLR with CTCs to predict the prognosis of GC and CRC patients furthermore.

## Materials and Methods

### Patients

This was a retrospective study which included the patients with GC and CRC who have received therapies between August 2016 and October 2020. All patients were staged according to the 8th TNM-classification (AJCC). The included criteria of the group of patients were as follows: 1) patients were aged from 18 to 85 years; 2) patients had the whole clinicopathological data and follow-up data; 3) the blood examination and CTCs counts detection were carried out before the postoperative antitumor treatment, such as chemotherapy or other therapies; 4) patients were diagnosed definitely by histopathology; 5) patients who were excluded were those who had chronic infection or acute infection in the process of tumor treatment confirmed by microbiology; 6) patients who were excluded were those who were under the treatment with glucocorticoids in a week.

### The Detection of CTCs

Approximately 5 ml peripheral blood was collected from every patient with GC or CRC before the treatment. The detection of CTCs, in brief, was used by immunomagnetic bead negative enrichment combined with immunofluorescence *in situ* hybridization (Im-FISH), according to our previous study (17).

### Statistical Analysis

The demographic data and clinicopathologic features were collected retrospectively. DFS was defined as the time from the date of surgery to the date of definite diagnosis of disease recurrence. OS was calculated from the date of treatment to the date of death or the last follow-up. The data was analyzed by using SPSS 22.0 software (IBM, NY, USA), and the graphs were profiled by using GraphPad Prism 6.0 (GraphPad Software, CA, USA). We constructed the receiver-operating-curve (ROC) by using the OS as the status variable and calculating the optimal cut-off value for NLR and CTCs by Youden index (sensitivity + specificity − 1). In order to demonstrate the relationship between NLR and clinicopathological data, Pearson’s chi-square test or Fisher exact test was used to analyze them. The same method was applied in testifying the association of CTCs and patients’ characteristics. Survival curves were calculated with the Kaplan–Meier analysis and compared with log-rank test. The multivariate analysis was used to evaluate the independent factors of prognosis. The P-value <0.05 was considered significant in this paper.

## Results

### The Characteristics of Patients

We enrolled 72 patients with gastrointestinal cancer from August 2016 to October 2020. Patients were followed up to October 2020. The median follow-up time was 50.18 ± 3.41 months. The characteristics of these 72 patients are shown in **Table 1**. The median age of all these patients was 65 years (range 37 to 85 years). For primary tumor location, there were 25 (34.7%) patients in colon, 19 (26.4%) in rectum and 28 (38.9%) in stomach, respectively. Among them, 15 patients (20.8%) had visceral metastasis such as pulmonary or hepatic metastasis. The connection of NLR and clinicopathological variables was performed in **Table 2**; meanwhile, the association of CTCs and characteristics was also shown in **Table 2**. Our univariate analysis revealed that TNM stage, T stage and the number of metastatic tumor sites were related to DFS (**Table 3**). For OS, TNM stage, T stage and the number of metastatic tumor sites had significance (**Table 3**). In consideration of the difference of N stage in gastric cancer and colorectal cancer, we analyzed the N stage with the survival separately, after which we found that N stage of colorectal cancer was related to DFS and OS, whereas in gastric cancer, N stage had significance in DFS but had no significance in OS (**Table 3**). Nevertheless, age, gender, primary tumor location, tumor differentiation had no significance of DFS and OS (**Table 3**). In addition, patients diagnosed with visceral metastasis had worse prognosis than patients with non-visceral metastasis in DFS and OS (**Table 3**).

**Table 1:** Clinicopathological variables of patients with gastrointestinal cancer.

Variables	n	%
Age		
≤65	37	51.4
>65	35	48.6
Gender		
Female	23	31.9
Male	49	68.1
Location		
Stomach	28	38.9
Colon	25	34.7
Rectum	19	26.4
Stage		
I–III	50	69.4
IV	22	30.6
T stage		
T1–T3	31	43.1
T4	41	56.9
N stage		
Stomach		
N0	8	11.1
N1–N3	20	27.8
Colorectal		
N0	18	25
N1–N2	26	36.1
Differentiation		
Poor	29	40.3
Well	43	59.7
Metastatic sites		
0	26	36.1
1	28	38.9
2/3	18	25
NLR		
≤3.21	50	69.4
>3.21	22	30.6
CTCs		
≤1	20	27.8
>1	52	72.2

**Table 2:** Association of NLR (>3.21 versus ≤3.21) or CTC (>1 versus ≤1) with clinicopathological variables.

Variables	NLR ≤ 3.21	NLR > 3.21	P value	CTC ≤ 1	CTC > 1	P value
	N = 50 (%)	N = 22 (%)		N = 20 (%)	N = 52 (%)	
Age			0.722			0.501
≤65	25 (50)	12 (54.5)		9 (45)	28 (53.8)	
>65	25 (50)	10 (45.5)		11 (55)	24 (46.2)	
Gender			0.165			0.73
Female	19 (38)	4 (18.2)		7 (35)	16 (30.8)	
Male	31 (62)	18 (81.8)		13 (65)	36 (69.2)	
Location			0.896			0.246
Stomach	19 (38)	9 (40.9)		7 (35)	21 (40.4)	
Colon	17 (34)	8 (36.4)		5 (25)	20 (38.5)	
Rectum	14 (28)	5 (22.7)		8 (40)	11 (21.1)	
TNM stage			<0.001^*^			0.001^*^
I–III	42 (84)	8 (36.4)		20 (100)	30 (57.7)	
IV	8 (16)	14 (63.6)		0 (0)	22 (42.3)	
T stage			0.021^*^			0.004^*^
T1–T3	26 (52)	5 (22.7)		14 (70)	17 (32.7)	
T4	24 (48)	17 (77.3)		6 (30)	35 (67.3)	
N stage						
Stomach			0.337			NA
N0	7 (36.8)	1 (11.1)		2 (28.6)	6 (28.6)	
N1–N3	12 (63.2)	8 (88.9)		5 (71.4)	15 (71.4)	
Colorectum			0.01^*^			0.071
N0	17 (54.8)	1 (7.7)		8 (61.5)	10 (32.3)	
N1–N2	14 (45.2)	12 (92.3)		5 (38.5)	21 (67.7)	
Differentiation			0.265			0.056
Poor	18 (36)	11 (50)		4 (20)	25 (48.1)	
Well	32 (64)	11 (50)		16 (80)	27 (51.9)	
Metastatic sites			<0.001^*^			0.006^*^
0	22 (44)	3 (13.6)		11 (55)	14 (26.9)	
1	22 (44)	7 (31.8)		9 (45)	20 (38.5)	
2/3	6 (12)	12 (54.6)		0 (0)	18 (34.6)	
Visceral metastasis			0.001^*^			0.007^*^
Yes	5 (10)	10 (45.5)		0 (0)	15 (28.8)	
No	45 (90)	12 (54.5)		20 (100)	37 (71.2)	

*P < 0.05; NA, not applicable.

**Table 3:** Univariate analysis of clinicopathological characteristics associated with survival.

Variables	DFS			OS		
	HR	95% CI	P value	HR	95% CI	P value
Age						
≤65	1.014	0.493–2.083	0.971	0.832	0.377–1.836	0.649
>65						
Gender						
Female	1.005	0.467–2.162	0.989	1.093	0.475–2.516	0.843
Male						
Location						
Stomach	0.813	0.511–1.293	0.382	0.899	0.549–1.471	0.672
Colon						
Rectum						
TNM stage						
I–III	6.202	2.959–13.001	<0.001*	6.986	3.004–16.249	<0.001*
IV						
T stage						
T1–T3	5.688	2.169–14.916	<0.001*	6.311	2.159–18.477	<0.001*
T4						
N stage						
Stomach						
N0	8.793	1.096–70.566	0.041*	6.34	0.788–51.009	0.083
N1–N3						
Colorectum						
N0	4.468	1.279–15.607	0.019^*^	12.68	1.665–96.568	0.014^*^
N1–N2						
Metastasis sites						
0	5.960	3.194–11.124	<0.001*	4.191	2.263–7.764	<0.001*
1						
2/3						
Visceral metastasis						
Yes	7.719	3.509–16.978	<0.001*	4.222	1.890–9.433	<0.001*
No						
Differentiation						
Poor	0.764	0.372–1.570	0.462	0.606	0.279–1.317	0.201
Well						
NLR						
≤3.21	6.561	3.126–13.774	<0.001^*^	7.214	3.173–16.401	<0.001^*^
>3.21						
CTC						
≤1	7.422	1.766–31.202	0.001^*^	14.186	1.904–105.697	0.001^*^
>1						

*P < 0.05; HR, Hazard ratio; CI, Confidence interval; DFS, Disease-free survival; OS, overall survival.

### The Relationship Between NLR With Clinicopathological Parameters and the Survival

We constructed the ROC by using the OS as the status variable (area under the curve (AUC) = 0.759) and determined the optimal cut-off value for NLR as 3.21. Patients were divided into two groups according to the cut-off value. In these patients, 22 (30.6%) of them had NLR>3.21 and the other 50 (69.4%) of patients had NLR ≤3.21. The association between NLR and clinicopathological variables was shown in **Table 2**. The results turned out that NLR was related with TNM stage, T stage, the number of metastatic tumor sites and visceral metastasis, whereas NLR seemingly had no significance with age, gender, primary tumor location and tumor differentiation. Among them, due to the difference of N stage of GC and CRC, we found that NLR was correlated with N stage of colorectal cancer but had no significance in N stage of gastric cancer.

**Figure 1:**
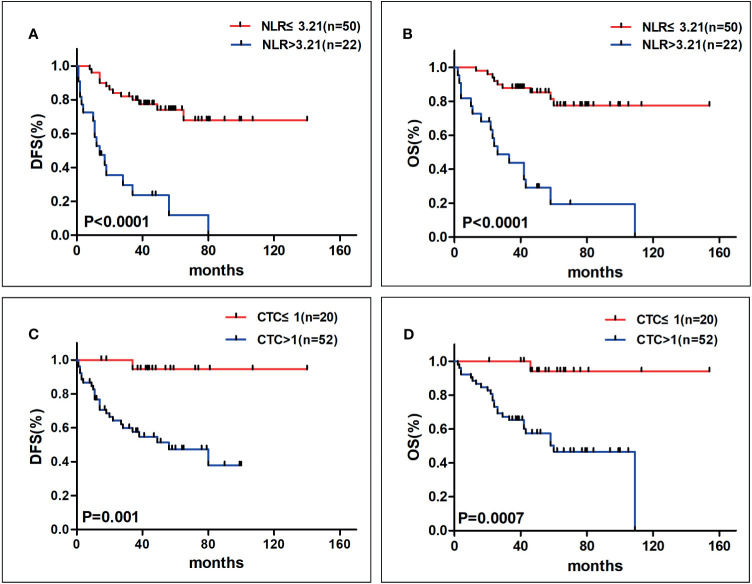
The Kaplan–Meier analysis curves for NLR on DFS **(A)** and OS **(B)** and for CTCs on DFS **(C)** and OS **(D)** (DFS, disease-free survival; OS, overall survival; NLR, neutrophil–lymphocyte ratio; CTCs, circulating tumor cells). Patients in higher NLR group had poorer prognosis with shorter DFS and OS, meanwhile, patients with more CTCs tended to have worse clinical outcome with shorter DFS and OS.

In these 72 patients, 30 of them developed tumor recurrence during the follow-up. Results from our data showed that 17 patients (56.7%) in high NLR group and 13 patients (43.3%) in low NLR group had recurrence. Otherwise, we observed the prognostic value of NLR in these two groups. We found that the patients with NLR ≤3.21 had longer DFS (median, not reached (NR) *vs*. 14 months, P <0.0001, **Figure 1A**) and OS (median, NR *vs*. 26 months, P <0.0001, **Figure 1B**) than patients with NLR >3.21. Furthermore, in order to estimate the relevance between NLR and survival, we got information from describing Kaplan–Meier curves and then discovered that the 5-year DFS rate of patients with NLR >3.21 was 11.9% and 74% of patients with NLR ≤3.21, respectively (P <0.0001). In terms of OS, the 5-year OS rate was 19.5% in higher NLR group and 77.5% in lower NLR group (P <0.0001).

### Association of CTCs With Clinicopathological Variables and Survival

As for CTCs, we constructed the ROC by using the OS as the status variable (AUC = 0.738) and found the suitable cut-off value for CTCs was 1. We assessed the relationship between CTCs and patients’ clinical outcome. The CTCs counts were ≤1 among 52 (72.2%) patients and >1 among 20 (27.8%) patients. The relationship between CTCs and characteristics was shown in **Table 2**. From our results, TNM stage, T stage, the number of metastatic sites and visceral metastasis were linked with CTCs. Unfortunately, CTCs had no significance with other variables, as age, gender, primary tumor location, N stage and tumor differentiation.

Among those patients who had recurrence, there were two patients (10%) in the CTC ≤1 group while 18 patients (90%) in the CTC >1 group. In this paper, we investigated whether CTCs could predict the clinical outcomes of gastrointestinal cancer. In the process of studying the prognostic value of CTCs, we found that individuals with more CTCs tended to have shorter DFS (median, NR *vs*. 49 months, P = 0.001, **Figure 1C**) and OS (median, NR *vs*. 60 months, P = 0.001, **Figure 1D**) than the patients with less CTCs. Moreover, we explored the relationship of CTCs and survival by using Kaplan–Meier analysis, which demonstrated that the 5-year DFS rate was 44.6% and 89.5% (P = 0.001), in higher CTCs and lower CTCs group, respectively. Meanwhile, patients with CTC>1 had a decreased 5- year OS rate than the group of CTC ≤1 (46.7% *vs*. 94.1%, P = 0.001).

### The Relationship Between NLR and CTCs Counts and Survival

**Figure 2:**
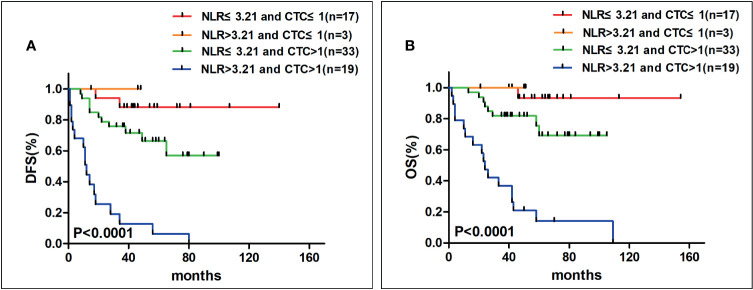
The Kaplan–Meier analysis curves on DFS **(A)** and OS **(B)** when associating NLR and CTCs counts (DFS, disease-free survival; OS, overall survival; NLR, neutrophil–lymphocyte ratio; CTCs, circulating tumor cells). In patients with higher NLR and higher CTCs counts had shorter DFS and OS.

Finally, our ultimate aim was to explore the combined effect of associating NLR with CTCs to predict the prognosis of gastrointestinal cancer. Therefore, we divided our patients into four groups: 1) patients with NLR ≤3.21 and CTC ≤1 (n = 17); 2) patients with NLR >3.21 and CTC ≤1 (n = 3); 3) patients with NLR ≤3.21 and CTC >1 (n = 33); and 4) patients with NLR >3.21 and CTC >1 (n = 19). After that we found that in the people who had both higher NLR and higher CTCs counts had shorter DFS (P <0.0001, **Figure 2A**) and OS (P <0.0001, **Figure 2B**) than the others.

## Discussion

Gastrointestinal cancer is one of the most common tumors among the world, which nowadays accompanies with high incidence. The incidence rate of GC and CRC were approximately 11.1/100,000 and 19.7/100,000 worldwide, respectively (19). In the last decades, the appearance of an endoscope has brought the great advancement of disease diagnosis. GC screening such as endoscopy and Barium imaging could improve the detection of early GC, which could improve the 5-year survival rate as well (20). Colonoscopy could decrease the overall mortality by about 29% in CRC (21). However, the survival of gastrointestinal cancer is still poor. Thus, the relatively exact biomarker to predict the prognosis of GC and CRC is necessary.

Currently, the vital role of inflammation in the tumor microenvironment has been found in different researches. It is worth noting that neutrophils and lymphocytes as indispensable components of inflammation which demonstrated that they may have an effect on the development of tumor. Neutrophils are the first line of defense of the body which can increase rapidly when body gets acute infection, various poisoning and the damage of tissue. Now, a lot of growing research has suggested the positive role of neutrophils in the growth of tumor (22–24). Neutrophils are major inflammatory constituents of TME, which can facilitate the growth and development of tumor by different mechanisms (24, 25). Diefenhardt et al. (26) found that leukocytosis and neutrophilia tended to predict the poor clinical outcome. Meanwhile, the variation of lymphocytes often related to virus infection, as the center of immune response. For the prognostic value of lymphocytes, De Giorgi et al. (27) concluded that lymphocytopenia can be regarded as an independent prognostic factor for metastatic breast cancer. Therefore, to some extent, NLR reflects a balance between the inflammation and antitumor immunity (28), but the breaking of balance would promote the inflammatory response and consequently result in tumor progression. Therefore, the value of NLR, as an inflammatory index, might be applied to predict the prognosis of GC and CRC, which have been investigated in previous papers. There was a meta-analysis by Sun et al. (29) suggesting that patients with higher NLR had poorer prognosis for OS and PFS than patients with normal NLR in gastric cancer. Similar results were concluded from a meta-analysis by Randy et al. (30) in gastrointestinal cancer. According to some studies, comparable results were reported, from which it was found that for patients with GC, high postoperative NLR was a favorable tool for indicating poor prognosis (31, 32). A systemic review by Haram et al. (33) revealed that pre-operative elevated NLR was related with shorter survival in both patients with localized CRC and with liver metastasis. Identically, in a retrospective analysis, Wu et al. (34) concluded that NLR was a crucial prognostic factor for OS and PFS in patients with CRC, which was a predictor for therapy response as well. The combination of NLR and PLR were regarded as a predictor for prognosis in GC (35), the same conclusion was presented among the gastrointestinal cancer as well by Nora et al. (36). In our article, patients with lower NLR had a better outcome with longer DFS and OS, which revealed that lower NLR tended to accompany with better prognosis. Favorably, our results from multivariate analysis indicated that NLR was an independent factor predicting the prognosis (**Table 4**).

**Table 4:** Independent factors associated with DFS and OS in multivariate analysis.

Variables	DFS			OS		
	HR	95% CI	P value	HR	95% CI	P value
Age	1.305	0.516–3.301	0.573	0.548	0.199–1.511	0.245
Gender	0.511	0.196–1.332	0.169	0.559	0.200–1.560	0.267
Location	0.687	0.258–1.834	0.454	0.688	0.241–1.965	0.485
TNM stage	1.692	0.572–5.008	0.342	2.382	0.787–7.208	0.124
T stage	2.117	0.957–4.682	0.064	2.940	1.224–7.063	0.016^*^
N stage	0.654	0.153–2.799	0.568	8.891	0.959–82.455	0.054
Metastatic sites	3.228	1.306–7.977	0.011^*^	0.728	0.246–2.154	0.566
NLR	3.425	1.223–9.593	0.019^*^	3.384	1.066–10.741	0.039^*^
CTC	2.841	0.594–13.598	0.191	4.800	0.562–41.009	0.152

*P < 0.05; HR, Hazard ratio; CI, Confidence interval; DFS, Disease-free survival; OS, overall survival.

CTCs are a kind of tumor cells which survive in the bloodstream, playing a significant role in the tumor metastatic process. Nowadays, the prognostic role of CTCs in the gastrointestinal cancer is under active investigation. In colon cancer, high CTCs were an independent factor which meant worse OS and DFS (16). As our previous study mentioned, in advanced colorectal cancer, patients with CTCs ≥3 were reported with unfortunate survival with shorter PFS and OS (17). Likewise, CTCs were considered as a predicting factor for PFS and OS in metastatic colorectal cancer according to other researches (37, 38). Gao et al. (15) summarized a meta-analysis indicating that in patients with GC, the detection of increased CTCs predicted poor prognosis. Our data show a definite consequence that patients with more CTCs had a poorer prognosis along with shorter OS and DFS as well. However, resulting from our multivariate analysis, CTCs might not be an independent factor for prognosis (**Table 4**), which may due to the fact that our follow-up period was not long enough and thus our ending events were not adequate.

As mentioned previously, many studies have found that single NLR or single CTCs had the prognostic role in GC and CRC. Particularly, our past study found that inflammatory index had significance in head and neck squamous cell carcinoma. Meanwhile, CTCs were demonstrated to have the prognostic value in head and neck squamous cell carcinoma and advanced colorectal cancer. Thus, in consideration of NLR being closely linked with tumor progression and CTCs having the capability to predict prognosis, we chose a direction of combining NLR with CTCs to predict the outcome of gastrointestinal cancer. There were several similar studies that combined inflammatory indices with CTCs to predict the prognosis in malignancies. De Giorgi et al. (11) conducted a work in patients with breast cancer that combined MLR with CTCs, which suggested that MLR and CTCs are independent roles together for prognosis. Hu Bo et al. (39) carried on a research in hepatocellular carcinoma, which integrated SII with CTCs to predict the prognosis, finding that patients with higher SII and detectable CTCs tended to have poor prognosis. Fortunately, from our own perspective, we obtained a result that in patients with gastrointestinal cancer who had both NLR >3.21 and CTC >1 had obviously shorter DFS and OS than those with NLR ≤3.21 and CTC ≤1, which indicated that combining the NLR and CTCs had great prognostic value of GC and CRC.

However, this research had several potential limitations. First, the samples size of our research was too small to find the prognostic value of NLR and CTCs counts, and the follow-up time was not long enough. Second, this research was a retrospective analysis; some data about patients may have unavoidable error. Thirdly, we only collected the peripheral blood and CTCs counts before chemotherapy or other therapy and we did not evaluate NLR and/or CTCs before and after chemotherapy or other therapy dynamically. Additionally, although CTCs had a different application for tumor prognosis, it is still difficult for CTCs to become common in the clinical practice because at present, the majority of CTCs detection is through epithelial markers (such as EpCAM and cytokeratins) and epithelial cell surface-associated glycoproteins (such as MUC-1) (1), whereas nowadays these methods tend to ignore CTCs with mesenchymal phenotypes. However, epithelial–mesenchymal transition can appear in the metastatic process of tumor, which means CTCs detection with mesenchymal markers may be more accurate (1).

Nowadays, a definite diagnosis for progression of cancer depends on the pathological examination of surgery or biopsy tissue. Traditional biopsy such as surgical and puncture biopsy tend to accompany with relatively major trauma and limiting sampling location. In this way, liquid biopsy, as the novel detecting method, has far less damage to human’s body than traditional biopsy (40). And in the subsequent process of tumor therapies, liquid biopsy may play a vital role in guiding treatment dynamically. For example, Tammingam et al. (41) divided patients with small lung cancer into different groups on the basis of CTCs positive/negative and chemotherapy/immunotherapy, of which the survival consequence showed that CTCs positive patients have clearly lower survival rate than the opposite groups. As a result, CTCs counts might be regarded as a tool to be applied to stratify patients into different groups and then direct treatment to a certain degree in the future.

## Conclusion

In conclusion, we found that higher NLR was an independent biomarker for poor prognosis in gastrointestinal cancer patients, while CTCs counts need further studies to be regarded as a significant factor. Meanwhile, combining NLR and CTCs counts might predict the clinical outcome in gastrointestinal cancer as well.

## Data Availability Statement

The original contributions presented in the study are included in the article/supplementary material. Further inquiries can be directed to the corresponding authors.

## Ethics Statement

The studies involving human participants were reviewed and approved by the ethics committee of Shanghai Ninth People’s Hospital affiliated to Shanghai Jiao Tong University School of Medicine.

## Author Contributions

HY and FL designed the study. XH, JW, and FL collected the samples. BJ, WZ, and HY followed up the overall survival and disease-free survival of the patients. BJ, HY, WZ, RC and CQ analyzed the data. CQ, RC and HY wrote the manuscript with assistance from BJ and YZ. All authors contributed to the article and approved the submitted version.

## Funding

This study was funded by Clinical Research Program of 9th People’s Hospital, Shanghai Jiao Tong University School of Medicine (JYLJ201808).

## Conflict of Interest

The authors declare that the research was conducted in the absence of any commercial or financial relationships that could be construed as a potential conflict of interest.

## References

[1] Thanh Huong P Gurshaney S Thanh Binh N Gia Pham A Hoang Nguyen H Thanh Nguyen X. Emerging Role of Circulating Tumor Cells in Gastric Cancer. Cancers (Basel) (2020) 12(3):695. 10.3390/cancers12030695 PMC7140068 32183503

[2] Aoyama T Oba K Honda M Sadahiro S Hamada C Mayanagi S. Impact of Postoperative Complications on the Colorectal Cancer Survival and Recurrence: Analyses of Pooled Individual Patients’ Data From Three Large Phase III Randomized Trials. Cancer Med (2017) 6(7):1573–80. 10.1002/cam4.1126 PMC5504309 28639738

[3] Gangireddy VGR Coleman T Kanneganti P Talla S Annapureddy AR Amin R. Polypectomy Versus Surgery in Early Colon Cancer: Size and Location of Colon Cancer Affect Long-Term Survival. Int J Colorectal Dis (2018) 33(10):1349–57. 10.1007/s00384-018-3101-z 29938362

[4] Anderson NM Simon MC. The Tumor Microenvironment. Curr Biol (2020) 30(16):R921–R5. 10.1016/j.cub.2020.06.081 PMC8194051 32810447

[5] Guo W Lu X Liu Q Zhang T Li P Qiao W. Prognostic Value of Neutrophil-to-Lymphocyte Ratio and Platelet-to-Lymphocyte Ratio for Breast Cancer Patients: An Updated Meta-Analysis of 17079 Individuals. Cancer Med (2019) 8(9):4135–48. 10.1002/cam4.2281 PMC6675722 31197958

[6] Kacan T Babacan NA Seker M Yucel B Bahceci A Eren AA. Could the Neutrophil to Lymphocyte Ratio be a Poor Prognostic Factor for non Small Cell Lung Cancers? Asian Pac J Cancer Prev (2014) 15(5):2089–94. 10.7314/apjcp.2014.15.5.2089 24716939

[7] Li S-H Lai H-L Tang Y Chien C-Y Fang F-M Huang T-L. Neutrophil Lymphocyte Ratio Is an Independent Prognosticator in Patients With Locally Advanced Head and Neck Squamous Cell Carcinoma Receiving Induction Chemotherapy With Docetaxel, Cisplatin, and Fluorouracil. J Cancer Res Pract (2019) 6(4):170–8. 10.4103/jcrp.Jcrp_12_19

[8] Gonda K Shibata M Sato Y Washio M Takeshita H Shigeta H. Elevated Neutrophil-to-Lymphocyte Ratio Is Associated With Nutritional Impairment, Immune Suppression, Resistance to S-1 Plus Cisplatin, and Poor Prognosis in Patients With Stage IV Gastric Cancer. Mol Clin Oncol (2017) 7(6):1073–8. 10.3892/mco.2017.1438 PMC5740823 29285377

[9] Sun Y Zhang Y Huang Z Lin H Lu X Huang Y. Combination of Preoperative Plasma Fibrinogen and Neutrophil-To-Lymphocyte Ratio (the F-NLR Score) as a Prognostic Marker of Locally Advanced Rectal Cancer Following Preoperative Chemoradiotherapy. World J Surg (2020) 44(6):1975–84. 10.1007/s00268-020-05407-3 32020327

[10] Walsh SR Cook EJ Goulder F Justin TA Keeling NJ. Neutrophil-Lymphocyte Ratio as a Prognostic Factor in Colorectal Cancer. J Surg Oncol (2005) 91(3):181–4. 10.1002/jso.20329 16118772

[11] De Giorgi U Mego M Scarpi E Giordano A Giuliano M Valero V. Association Between Circulating Tumor Cells and Peripheral Blood Monocytes in Metastatic Breast Cancer. Ther Adv Med Oncol (2019) 11:1758835919866065. 10.1177/1758835919866065 31452692 PMC6696837

[12] Castello A Carbone FG Rossi S Monterisi S Federico D Toschi L. Circulating Tumor Cells and Metabolic Parameters in NSCLC Patients Treated With Checkpoint Inhibitors. Cancers (Basel) (2020) 12(2):487. 10.3390/cancers12020487 PMC7072667 32092983

[13] Jatana KR Balasubramanian P Lang JC Yang L Jatana CA White E. Significance of Circulating Tumor Cells in Patients With Squamous Cell Carcinoma of the Head and Neck: Initial Results. Arch Otolaryngol Head Neck Surg (2010) 136(12):1274–9. 10.1001/archoto.2010.223 PMC3740520 21173379

[14] Zhou S Wang L Zhang W Liu F Zhang Y Jiang B. Circulating Tumor Cells Correlate With Prognosis in Head and Neck Squamous Cell Carcinoma. Technol Cancer Res Treat (2021) 20:1533033821990037. 10.1177/1533033821990037 33641530 PMC7924006

[15] Gao Y Xi H Wei B Cui J Zhang K Li H. Association Between Liquid Biopsy and Prognosis of Gastric Cancer Patients: A Systematic Review and Meta-Analysis. Front Oncol (2019) 9:1222. 10.3389/fonc.2019.01222 31850190 PMC6901923

[16] Hinz S Hendricks A Wittig A Schafmayer C Tepel J Kalthoff H. Detection of Circulating Tumor Cells With CK20 RT-PCR Is an Independent Negative Prognostic Marker in Colon Cancer Patients - a Prospective Study. BMC Cancer (2017) 17(1):53. 10.1186/s12885-016-3035-1 28086834 PMC5237158

[17] Wang L Zhou S Zhang W Wang J Wang M Hu X. Circulating Tumor Cells as an Independent Prognostic Factor in Advanced Colorectal Cancer: A Retrospective Study in 121 Patients. Int J Colorectal Dis (2019) 34(4):589–97. 10.1007/s00384-018-03223-9 30627849

[18] Zhou S Yuan H Wang J Hu X Liu F Zhang Y. Prognostic Value of Systemic Inflammatory Marker in Patients With Head and Neck Squamous Cell Carcinoma Undergoing Surgical Resection. Future Oncol (2020) 16(10):559–71. 10.2217/fon-2020-0010 32166977

[19] Feng RM Zong YN Cao SM Xu RH. Current Cancer Situation in China: Good or Bad News From the 2018 Global Cancer Statistics? Cancer Commun (Lond) (2019) 39(1):22. 10.1186/s40880-019-0368-6 31030667 PMC6487510

[20] Khanderia E Markar SR Acharya A Kim Y Kim YW Hanna GB. The Influence of Gastric Cancer Screening on the Stage at Diagnosis and Survival: A Meta-Analysis of Comparative Studies in the Far East. J Clin Gastroenterol (2016) 50(3):190–7. 10.1097/mcg.0000000000000466 26844858

[21] Singh H Nugent Z Demers AA Kliewer EV Mahmud SM Bernstein CN. The Reduction in Colorectal Cancer Mortality After Colonoscopy Varies by Site of the Cancer. Gastroenterology (2010) 139(4):1128–37. 10.1053/j.gastro.2010.06.052 20600026

[22] Huang Q Diao P Li CL Peng Q Xie T Tan Y. Preoperative Platelet-Lymphocyte Ratio Is a Superior Prognostic Biomarker to Other Systemic Inflammatory Response Markers in non-Small Cell Lung Cancer. Med (Baltimore) (2020) 99(4):e18607. 10.1097/MD.0000000000018607 PMC7004654 31977852

[23] Tazeen S Prasad K Harish K Sagar P Kapali AS Chandramouli S. Assessment of Pretreatment Neutrophil/Lymphocyte Ratio and Platelet/Lymphocyte Ratio in Prognosis of Oral Squamous Cell Carcinoma. J Oral Maxillofac Surg (2020) 78(6):949–60. 10.1016/j.joms.2020.01.001 32027861

[24] Szilasi Z Josa V Zrubka Z Mezei T Vass T Merkel K. Neutrophil-To-Lymphocyte and Platelet-To-Lymphocyte Ratios as Prognostic Markers of Survival in Patients With Head and Neck Tumours-Results of a Retrospective Multicentric Study. Int J Environ Res Public Health (2020) 17(5):1742. 10.3390/ijerph17051742 PMC7084240 32155982

[25] Wu L Saxena S Singh RK. Neutrophils in the Tumor Microenvironment. Adv Exp Med Biol (2020) 1224:1–20. 10.1007/978-3-030-35723-8_1 32036601 PMC7325741

[26] Diefenhardt M Hofheinz RD Martin D Beißbarth T Arnold D Hartmann A. Leukocytosis and Neutrophilia as Independent Prognostic Immunological Biomarkers for Clinical Outcome in the CAO/ARO/AIO-04 Randomized Phase 3 Rectal Cancer Trial. Int J Cancer (2019) 145(8):2282–91. 10.1002/ijc.32274 30868576

[27] De Giorgi U Mego M Scarpi E Giuliano M Giordano A Reuben JM. Relationship Between Lymphocytopenia and Circulating Tumor Cells as Prognostic Factors for Overall Survival in Metastatic Breast Cancer. Clin Breast Cancer (2012) 12(4):264–9. 10.1016/j.clbc.2012.04.004 22591634

[28] Fukuda N Wang X Ohmoto A Urasaki T Sato Y Nakano K. Sequential Analysis of Neutrophil-To-Lymphocyte Ratio for Differentiated Thyroid Cancer Patients Treated With Lenvatinib. In Vivo (2020) 34(2):709–14. 10.21873/invivo.11828 PMC7157845 32111774

[29] Sun J Chen X Gao P Song Y Huang X Yang Y. Can the Neutrophil to Lymphocyte Ratio Be Used to Determine Gastric Cancer Treatment Outcomes? A Systematic Review and Meta-Analysis. Dis Markers (2016) 2016:7862469. 10.1155/2016/7862469 26924872 PMC4746375

[30] Bowen RC Little NAB Harmer JR Ma J Mirabelli LG Roller KD. Neutrophil-To-Lymphocyte Ratio as Prognostic Indicator in Gastrointestinal Cancers: A Systematic Review and Meta-Analysis. Oncotarget (2017) 8(19):32171–89. 10.18632/oncotarget.16291 PMC5458276 28418870

[31] Li Z Li S Ying X Zhang L Shan F Jia Y. The Clinical Value and Usage of Inflammatory and Nutritional Markers in Survival Prediction for Gastric Cancer Patients With Neoadjuvant Chemotherapy and D2 Lymphadenectomy. Gastric Cancer (2020) 23(3):540–9. 10.1007/s10120-019-01027-6 PMC7165147 32072387

[32] Miyamoto R Inagawa S Sano N Tadano S Adachi S Yamamoto M. The Neutrophil-to-Lymphocyte Ratio (NLR) Predicts Short-Term and Long-Term Outcomes in Gastric Cancer Patients. Eur J Surg Oncol (2018) 44(5):607–12. 10.1016/j.ejso.2018.02.003 29478743

[33] Haram A Boland MR Kelly ME Bolger JC Waldron RM Kerin MJ. The Prognostic Value of Neutrophil-to-Lymphocyte Ratio in Colorectal Cancer: A Systematic Review. J Surg Oncol (2017) 115(4):470–9. 10.1002/jso.24523 28105646

[34] Wu Y Li C Zhao J Yang L Liu F Zheng H. Neutrophil-To-Lymphocyte and Platelet-to-Lymphocyte Ratios Predict Chemotherapy Outcomes and Prognosis in Patients With Colorectal Cancer and Synchronous Liver Metastasis. World J Surg Oncol (2016) 14(1):289. 10.1186/s12957-016-1044-9 27852294 PMC5112720

[35] Hirahara T Arigami T Yanagita S Matsushita D Uchikado Y Kita Y. Combined Neutrophil-Lymphocyte Ratio and Platelet-Lymphocyte Ratio Predicts Chemotherapy Response and Prognosis in Patients With Advanced Gastric Cancer. BMC Cancer (2019) 19(1):672. 10.1186/s12885-019-5903-y 31286873 PMC6615151

[36] Nora I Shridhar R Huston J Meredith K. The Accuracy of Neutrophil to Lymphocyte Ratio and Platelet to Lymphocyte Ratio as a Marker for Gastrointestinal Malignancies. J Gastrointest Oncol (2018) 9(5):972–8. 10.21037/jgo.2018.08.05 PMC6219984 30505600

[37] Groot Koerkamp B Rahbari NN Buchler MW Koch M Weitz J. Circulating Tumor Cells and Prognosis of Patients With Resectable Colorectal Liver Metastases or Widespread Metastatic Colorectal Cancer: A Meta-Analysis. Ann Surg Oncol (2013) 20(7):2156–65. 10.1245/s10434-013-2907-8 23456317

[38] Cohen SJ Punt CJ Iannotti N Saidman BH Sabbath KD Gabrail NY. Relationship of Circulating Tumor Cells to Tumor Response, Progression-Free Survival, and Overall Survival in Patients With Metastatic Colorectal Cancer. J Clin Oncol (2008) 26(19):3213–21. 10.1200/JCO.2007.15.8923 18591556

[39] Hu B Yang X-R Xu Y Sun Y-F Sun C Guo W. Systemic Immune-Inflammation Index Predicts Prognosis of Patients After Curative Resection for Hepatocellular Carcinoma. Clin Cancer Res (2014) 20(23):6212–22. 10.1158/1078-0432.Ccr-14-0442 25271081

[40] Kulasinghe A Hughes BGM Kenny L Punyadeera C. An Update: Circulating Tumor Cells in Head and Neck Cancer. Expert Rev Mol Diagn (2019) 19(12):1109–15. 10.1080/14737159.2020.1688145 31680565

[41] Tamminga M de Wit S Schuuring E Timens W Terstappen L Hiltermann TJN. Circulating Tumor Cells in Lung Cancer Are Prognostic and Predictive for Worse Tumor Response in Both Targeted- and Chemotherapy. Transl Lung Cancer Res (2019) 8(6):854–61. 10.21037/tlcr.2019.11.06 PMC6976367 32010564

